# Contraceptive rings promote vaginal lactobacilli in a high bacterial vaginosis prevalence population: A randomised, open-label longitudinal study in Rwandan women

**DOI:** 10.1371/journal.pone.0201003

**Published:** 2018-07-23

**Authors:** Tania Crucitti, Liselotte Hardy, Janneke van de Wijgert, Stephen Agaba, Jozefien Buyze, Evelyne Kestelyn, Thérèse Delvaux, Lambert Mwambarangwe, Irith De Baetselier, Vicky Jespers, for the Ring Plus study group

**Affiliations:** 1 Institute of Tropical Medicine, Antwerp, Belgium; 2 University of Liverpool, Liverpool, United Kingdom; 3 Rinda Ubuzima, Kigali, Rwanda; University of Alabama at Birmingham School of Medicine, UNITED STATES

## Abstract

**Background:**

Hormonal contraception has been associated with a reduced risk of vaginal dysbiosis, which in turn has been associated with reduced prevalence of sexually transmitted infections (STIs), including HIV. Vaginal rings are used or developed as delivery systems for contraceptive hormones and antimicrobial drugs for STI and HIV prevention or treatment. We hypothesized that a contraceptive vaginal ring (CVR) containing oestrogen enhances a lactobacilli-dominated vaginal microbial community despite biomass accumulation on the CVR’s surface.

**Methods:**

We enrolled 120 women for 12 weeks in an open-label NuvaRing^®^ study at Rinda Ubuzima, Kigali, Rwanda. Vaginal and ring microbiota were assessed at baseline and each ring removal visit by Gram stain Nugent scoring (vaginal only), quantitative PCR for *Lactobacillus* species, *Gardnerella vaginalis* and *Atopobium vaginae*, and fluorescent in situ hybridization to visualize cell-adherent bacteria. Ring biomass was measured by crystal violet staining.

**Results:**

Bacterial vaginosis (BV) prevalence was 48% at baseline. The mean Nugent score decreased significantly with ring use. The presence and mean log_10_ concentrations of *Lactobacillus* species in vaginal secretions increased significantly whereas those of *G*. *vaginalis* and presence of *A*. *vaginae* decreased significantly. Biomass accumulated on the CVRs with a species composition mirroring the vaginal microbiota. This ring biomass composition and optical density after crystal violet staining did not change significantly over time.

**Conclusions:**

NuvaRing^®^ promoted lactobacilli-dominated vaginal microbial communities in a population with high baseline BV prevalence despite the fact that biomass accumulated on the rings.

## Introduction

The vaginal microbial community (VMC) of healthy women consists predominantly of *Lactobacillus* spp [1,2]. Its diversity increases in the context of vaginal dysbiosis, which is most commonly consistent with the well-known clinical condition bacterial vaginosis (BV) [3,4]. Associations between the presence of lactobacilli-dominated VMC and lower prevalence of sexually transmitted infections (STIs), including HIV, have been shown [5]. Similarly, vaginal dysbiosis has been reported to be associated with the presence of STIs [6] and to increase the risk of *Chlamydia trachomatis*, *Neisseria gonorrhoea*, *Trichomonas vaginalis*, *Mycoplasma genitalium*, human papillomavirus, herpes simplex virus type 2 (HSV-2) and HIV infection [7–13].

Lactobacilli ferment glucose into lactic acid, which results in an acidic vaginal environment. This is considered to be key to rendering the vagina unfavourable for STI pathogens and anaerobic bacteria associated with BV [14,15]. In addition, lactobacilli produce bacteriocins and compete with STI pathogens for nutrients and vaginal surface epithelium to adhere to [16]. Interventions aimed at restoring and enhancing a lactobacilli-dominated VMC and reducing BV incidence may contribute to the global reduction of the STI and HIV burden. Several studies have shown that women using hormonal contraception are less likely to develop BV [17]. Oestrogen stimulates vaginal epithelial cells to produce glycogen, which is a source of the cell-free glucose that lactobacilli ferment [18].

Although a lactobacilli-dominated VMC is associated with vaginal health, not all lactobacilli are equally protective [16,19]. *Lactobacillus crispatus*, *L*. *iners*, *L*. *jensenii*, *L*. *gasseri* and *L*. *vaginalis* are the most common lactobacilli species found in the vagina [1,16]. Their occurrence varies according to exogenous and endogenous exposures such as age, sexual activity, vaginal oestrogen levels, and menses [14]. *L*. *crispatus* and *L*. *iners* are most frequently detected. *L*. *crispatus* offers protection against vaginal dysbiosis and STIs, whereas *L*. *iners*, although present in almost all women worldwide, has been associated with more frequent transition into a dysbiotic VMC [20,21] and correlates well with BV associated bacteria [22]. It still remains to be elucidated to what extent *L*. *gasseri*, *L*. *jensenii* and *L*. *vaginalis* protect the vaginal environment.

Recently, BV has been associated with the presence of biofilm on vaginal epithelial cells [23,24]. This phenomenon was originally observed by Amsel et al., who called the exfoliated vaginal epithelial cells covered with bacteria”clue cells” [25]. Biofilm presence in the vagina has since been confirmed using fluorescence in situ hybridization (FISH) techniques [23]. Studies have also shown biofilms on vaginally inserted inert materials such as intra-uterine devices and tampons [26–28], and more recently on vaginal rings [29–32].

Vaginal rings are currently available as contraceptive vaginal rings (CVRs), and are being developed as delivery systems for antimicrobial drugs to prevent or treat STIs, including HIV, (also named vaginal microbicides [33,34]) or for both hormones and antimicrobial drugs (also named multipurpose vaginal rings [35,36]). CVRs such as the NuvaRing^®^ are typically worn for three weeks followed by a one week break to allow for withdrawal bleeding. However, in order for a vaginal ring to prevent HIV or other STIs, it should be worn continuously over an extended period of time. As yet, not much is known about biomass development on vaginal rings. We identified three studies: a small study used rings loaded with tenofovir in primates [29], another studied vaginal rings releasing antiretrovirals for HIV prevention [30], and one reported biomass on vaginal rings delivering acyclovir for treatment and suppression of genital HSV [31,32]. Biomass was not detected on a Nuvaring^®^ used for three weeks by one healthy woman [37].

Overall, studies on the effect of potential vaginal ring biomass on the vaginal microbiota and on the extent to which the local release of oestrogen and biomass accumulation influence one another are lacking.

We have previously reported our findings regarding biomass deposition on the NuvaRing^®^ and its association with the status of the VMC. We also found that the concentration of *Gardnerella vaginalis* and *Atopobium vaginae* in the eluates of the vaginal ring fragments measured by quantitative (q)PCR were positively associated with the density of the biomass on the CVR [38].

In this study we longitudinally assessed the impact of NuvaRing^®^ use on the incidence of BV by Nugent score and on selected vaginal bacterial species and studied the presence of biofilm in the vagina and biomass on the ring in a population of Rwandan women with a high BV prevalence. We hypothesized that CVR use would promote a lactobacilli-dominated VMC even though biomass may accumulate on the CVRs.

## Methods

### Design

This study was an open-label single-centre cohort study evaluating the safety on the vaginal microbiota of the NuvaRing® (Organon, Oss, The Netherlands) in Rwandan women (ClinicalTrials.gov, identifier NCT01796613)(S1 Protocol, S1 Checklist) [39]. The study was conducted at the Rinda Ubuzima (RU) research centre in Kigali, Rwanda. HIV negative, non-pregnant women were randomised, using allocation sequence and envelopes created at the ITM, to an intermittent or continuous CVR use regimen. The randomisation allocation sequence and Women in the intermittent use group had the ring inserted for three weeks, followed by one week off; women in the continuous use group used the ring for three weeks, with the next ring being inserted immediately after the previous one and no off period in between. The women were followed for 12 weeks with study visits planned to coincide with times of ring removal and insertion.

The study was reviewed and approved by the ethics committees of the Institute of Tropical Medicine (ITM), Belgium; the University Hospital Antwerp, Belgium (approval number: B300201316845); the University of Liverpool, UK; the Rwandan National Ethics Committee; National Health Research Committee; and Ministry of Health. All study participants provided written informed consent for participation in the study.

### Investigational product

The NuvaRing® releases etonogestrel and ethinylestradiol at an average of 0.120 mg and 0.015 mg, respectively, per 24 hours, over three weeks.

### Study participants

We recruited healthy women who were not using a modern contraceptive method, and had not been using one in the three months prior to screening. Women eligible for enrolment were aged 18–35, currently not pregnant, sexually active, HIV negative, and not using antimicrobials [39].

### Clinical study

At baseline, blood was taken for HIV, HSV-2 and syphilis serology and urine for pregnancy testing. The urine pregnancy testing was repeated at ring removal visits; no pregnancies were detected during follow-up. Vaginal specimens were collected by a study physician at baseline and ring removal visits. In continuous users, samples were taken before the next ring insertion. Vaginal specimens were tested for *Chlamydia trachomatis* and *Neisseria gonorrhoeae* by PCR at baseline, for *Trichomonas vaginalis* and *Candida* spp. by wet mount and for BV by Nugent scoring of Gram stained slides at baseline and ring removal visits. We only employed validated test kits, methods, and procedures. Diagnosed infections were treated according to national guidelines, which meant that BV and candidiasis were only treated when symptomatic.

### Laboratory procedures

#### Vaginal specimens

During each pelvic examination, vaginal fluid from the posterior fornix of the vaginal wall was collected using one cotton and two Copan flocked® (Copan, Brescia, Italy) swabs. The cotton swab was used to prepare a wet mount, which was read within 20 minutes, and subsequent Gram staining and Nugent scoring [40]. In addition, the cotton swab was rolled onto a Superfrost Plus® slide (Menzel-Gläser, Braunschweig, Germany) for future FISH analysis. The slides for Nugent scoring and FISH were heat-fixed. The Superfrost Plus® slides were stored and shipped at room temperature to the ITM where they were re-fixed in Carnoy solution (6:3:1, ethanol:chloroform:glacial acetic acid) for at least 12 hours. The Copan flocked® swabs were eluted by vortexing each swab for a minimum of 15 seconds in 1.2 mL diluted phosphate buffered saline (dPBS) (pH 7.4–1:9, PBS:saline). The eluates were stored at -80°C until shipped frozen to the ITM for qPCR analysis.

The Superfrost Plus® slides were examined using Peptide Nucleic Acid (PNA)-FISH employing species-specific probes for *Atopobium vaginae* (AtoITM1) and *Gardnerella vaginalis* (Gard162), and the broad-range BacUni-1 probe. Procedures and definitions of observations are described elsewhere [41].

The total bacterial load in the vaginal specimens of *Lactobacillus* genus, *L*. *crispatus*, *L*. *iners*, *L*. *jensenii*, *L*. *gasseri*, *L*. *vaginalis*, *A*. *vaginae* and *G*. *vaginalis* was determined by qPCR assays. DNA was extracted from 250 μL of Copan® flocked swabs ‘eluates using the magnetic bead m2000 System DNA extraction kit on the Abbott m2000 automated platform (Abbott, Maidenhead, UK). The extraction program as customized by the manufacturer for DNA plasma extraction was optimized by including a pre-extraction heating lysis step. qPCR was performed for each bacterium species separately, as previously described [42].

#### Contraceptive vaginal rings

All used CVRs were collected by the study clinician and immediately delivered to the onsite RU laboratory. The CVRs were cut into three identical pieces using a ring template. One piece for PNA-FISH was stored in 3 mL Carnoy solution between 2–8°C; one piece for qPCR was kept in 3 mL dPBS at -80°C; and the third piece was submerged in 3 mL of glutaraldehyde, transferred after two weeks into 3 mL of formaldehyde and stored at 2–8°C, for crystal violet (CV) staining and scanning electron microscopy (SEM). Pieces in dPBS were shipped under frozen condition, the others at ambient temperature to the ITM. The ring pieces were processed and analysed as described elsewhere [38].

### Sample size and power calculation

The study sample size calculation was based on the primary objective to assess the before-after changes in the vaginal microbiota in both groups. To detect a change of 0.5 log_10_ in *Lactobacillus* genus concentration, expressed as genome equivalents (geq) per ml of vaginal eluate, after ring use within each randomization group with 95% power using a two-sided paired test with alpha = 5% and assuming a standard deviation of 1 log_10_, we required 52 women per group. To correct for early withdrawals and women lost-to-follow up, we randomised 120 women (60 randomised to the intermittent regimen, and 60 to the continuous regimen) to ensure we had 104 women with primary endpoint data available.

### Statistical analysis

Women in each treatment group were described with respect to baseline characteristics using medians and interquartile ranges (IQRs) for continuous characteristics and counts and percentages for categorical characteristics. The proportion of ring-use emergent BV cases (i.e., women with Nugent score 0–3 at enrolment and at least one Nugent score of 7–10 at any visit after first ring insertion) were compared using Fisher's exact test.

Bacteria present in the vaginal microbiota and/or in the ring biomass as determined by qPCR were expressed categorically as present or absent and as log_10_-transformed concentrations in geq/mL of vaginal swab eluate. Samples in which none of the assessed species were detected were excluded and considered inadequate (inhibition or insufficient sample material). A composite qPCR vaginal health score was calculated as log_10_ *Lactobacillus* genus concentration -log_10_ (*G*. *vaginalis*+*A*. *vaginae* concentrations) [43].

The evolutions of vaginal microbiota and ring biomass compositions by qPCR over time were modelled with linear or logistic mixed effects models using one different variance parameter per random effect with covariance 0. These models include fixed effects for time and the interaction between time and intervention group and random effects for intercept and time. For ring biomass data, baseline measurements were not applicable by definition. We compared the use of time as a categorical variable to a linear time effect (estimating difference in outcome between consecutive visits) and a binary time effect (estimating difference in outcome between post-baseline and baseline) and selected the most appropriate one for each model.

Time evolution of log_10_ geq of species/mL of vaginal swab eluate were only modelled if the species were present in at least 75% of samples for at least 50% of women.

Effects of intermittent versus continuous use were tested as differences in slopes in the models at the 5% level (two-sided).

We also planned to adjust for BV treatment within the last three weeks. However the majority of women with BV by Nugent were asymptomatic and did not require treatment according to the Rwandan national guidelines. Only four women received metronidazole treatment within the three weeks of specimen collection. A pilot analysis with adjustment provided similar results as the analyses without adjustment, and it was therefore decided not to proceed with the adjustment for BV treatment.

Analyses were performed using Stata, version 14.0 (StataCorp, College Station, TX, USA).

## Results

### Characteristics of the study population

**Fig 1:**
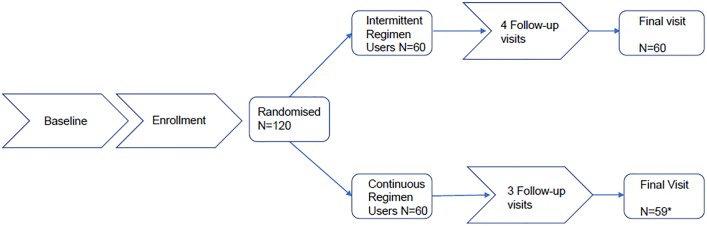
Participant flow. N = number of participants, * 1 discontinued after one cycle of vaginal ring use.

The study was conducted from June 2013 until January 2014. All 120 study participants, 60 intermittent and 60 continuous users, provided demographic and microbiological data at baseline, and 119 participants completed the study. One participant from the continuous use group discontinued prematurely and contributed only one cycle of CVR use (Fig 1). The median age was 28, about two thirds of women ever used a hormonal method of contraception, and almost all had at least one child (Table 1).

**Table 1:** Baseline characteristics, reproductive tract and sexually transmitted infections of all randomised participants who used at least one contraceptive vaginal ring.

	Intermittent Use	Continuous Use	Total
N	60	60	120
Age—**(year): median (IQR)**	28 (25.5, 31)	28.5 (26, 32)	28 (26, 31.5)
Highest Level of Education—**n (%)**			
**No schooling**	9 (15)	6 (10)	15 (13)
**Primary school not completed**	15 (25)	21 (35)	36 (30)
**Primary school completed**	24 (40)	20 (33)	44 (37)
**Secondary school not completed**	8 (13)	9 (15)	17 (14)
**Secondary school completed**	2 (3)	2 (3)	4 (3)
**More than secondary school**	2 (3)	2 (3)	4 (3)
Marital Status/Home Situation—**n (%)**			
**Married**	37 (62)	36 (60)	73 (61)
**Living together**	16 (27)	16 (27)	32 (27)
**Regular partner, but not living together**	7 (12)	8 (13)	15 (13)
Contraception History—**n (%)**			
**None**	19 (32)	22 (37)	41 (34)
Any modern method*	41 (68)	38 (63)	79 (66)
**Hormonal**			
**Injectables**^**1**^**- n (%)**	32 (53)	27 (45)	59 (49)
**Pills**^**1**^^,^^**3**^**- n (%)**	11 (18)	18 (30)	29 (24)
**IUD**^**1**^**- n (%)**	1 (2)	0 (0)	1 (1)
Pregnancies—**n (%)**			
**0**	2 (3)	3 (5)	5 (4)
**1**	12 (20)	10 (17)	22 (18)
**2**	22 (37)	17 (28)	39 (33)
**3 or more**	24 (40)	30 (50)	54 (45)
Any vaginal deliveries—**n (%)**	55 (92)	51 (85)	106 (88)
Any C-sections—**n (%)**	6 (10)	11 (18)	17 (14)
*C*. *trachomatis* PCR—positive **n (%)**	4 (7)	6 (10)	10 (8)
*N*. *gonorrhoea* PCR—positive **n (%)**	2 (3)	5 (8)	7 (6)
HIV serology—positive **n (%)**	0 (0)	0 (0)	0 (0)
HSV-2 serology—positive **n (%)**	21 (35)	26 (43)	47 (39)
Syphilis serology—positive **n (%)**	3 (5)	3 (5)	6 (5)
Wet Mount: *Candida* sp- positive **n (%)**	2 (3)	4 (7)	6 (5)
*T*. *vaginalis***—**positive **n (%)**	5 (8)	4 (7)	9 (8)
Clue Cells—**n (%)**			
**None**	44 (73)	37 (62)	81 (68)
**<20%**	9 (15)	16 (27)	25 (21)
**> = 20%**	7 (12)	7 (12)	14 (12)
Nugent score^2^**- n (%)**			
**0–3**	24 (40)	24 (41)	48 (40)
**4–6**	7 (12)	7 (12)	14 (12)
**7–10**	29 (48)	28 (47)	57 (48)

IQR: Interquartile Range

^1^More than one answer possible

^2^1 Missing/unreadable

^3^combined oral contraceptive pills

* None of the participants had ever used a vaginal ring for contraception prior to enrolment in the study

### High prevalence of BV and BV vaginal biofilms at baseline

A total of 119/120 vaginal slides collected at baseline for Nugent scoring were interpretable: 40% showed a normal vaginal microbiota; 12% an intermediate microbiota; and 48% BV (Table 1). Two women with BV had clinical symptoms and were treated.

The FISH analysis provided 100/120 interpretable results at baseline; 39% of the vaginal slides showed planktonic/dispersed bacteria only (morphologically indistinguishable, planktonic bacteria are free-living whereas dispersed bacteria are dispersed from a biofilm) and 17 of them did not show any *G*. *vaginalis* and *A*. *vaginae*. The other 61% of vaginal slides showed a biofilm and on nine of these slides no *G*. *vaginalis* and *A*. *vaginae* was detected as part of the biofilm. Planktonic/dispersed *G*. *vaginalis* was observed in 79% of the interpretable slides, of which 65% (51/79) included cell-adherent *G*. *vaginalis* in a biofilm structure. A total of 52% (52/100) of vaginal slides showed biofilm containing *G*. *vaginalis*. Planktonic/dispersed *A*. *vaginae* was observed in 55% of the interpretable slides, of which 49% (27/55) were cell-adherent. Finally, 28% (28/100) showed biofilm structures containing *A*. *vaginae* and all of them also contained *G*. *vaginalis*.

Of the 120 vaginal specimens analysed by qPCR, one could not be interpreted. *Lactobacillus* genus was detected in the majority (92%) of the vaginal specimens: *L*. *iners* was most frequently (75%) detected, followed by *L*. *vaginalis* (30%), *L*. *crispatus* (14%), *L*. *gasseri* (15%), and *L*. *jensenii* (13%). The presence of *G*. *vaginalis* (84%) and *A*. *vaginae* (61%) was also high.

### High incidence and persistence of BV

Of the 48 women with a Nugent score of 0–3 at baseline, 17% (4/24) of the intermittent and 33% (8/24) of the continuous users developed a Nugent score of 7–10 at least once during ring use; the difference between the two study groups was not significant. BV persisted or recurred during the study in 67% (38/57) of the women who had BV at baseline.

Over the course of the study, six women were treated with metronidazole: three for BV and three for trichomoniasis. At the final visit one extra woman was treated for BV and four for trichomoniasis.

### Lactobacilli presence and concentrations increased during ring use but the presence of a polymicrobial biofilm remained

The odds of presence of lactobacilli in vaginal secretions increased significantly over time within each group (intermittent: *P* = 0.031, continuous: *P* = 0.041) with no significant difference between the two study groups (Table 2, S1 Fig). The increases were significant over time for *L*. *crispatus*, *L*. *jensenii* and *L*. *vaginalis* (*P* value range 0.003–0.033) but not for *L*. *gasseri*. The increase of the odds of presence over time of *L*. *iners* was significant for the continuous use group (*P* = 0.027) and borderline significant for the intermittent use group (*P* = 0.051). The odds of *G*. *vaginalis* and *A*. *vaginae* presence decreased significantly over time within each group (*G*. *vaginalis* intermittent: *P* = 0.012, continuous: *P* = 0.004; *A vaginae* both groups: *P* = 0.001), with no significant difference between the groups.

**Table 2:** Longitudinal analysis of species presence in the vagina and in contraceptive vaginal ring biomasses in both study groups.

	Time evolution for intermittent use group		Time evolution for continuous use group		Difference in time evolution between groups	
	OR (95% CI)	*P*-value	OR (95% CI)	*P*-value	*P*-value	Time effect
**Vaginal specimen**						
***Lactobacillus* genus**	5.7 (1.2, 27.6)	0.031	4.4 (1.1, 18.0)	0.041	0.782	binary
***L*. *crispatus***	7.3 (1.5, 33.6)	0.014	4.5 (1.1, 17.5)	0.033	0.566	binary
***L*. *iners***	8.4 (1.0, 71.5)	0.051	13.1 (1.3, 128.3)	0.027	0.389	linear
***L*. *jensenii***	1.7 (1.1, 2.6)	0.022	1.8 (1.1, 2.8)	0.021	0.873	linear
***L*. *gasseri***	0.7 (0.3, 1.5)	0.363	1.1 (0.6, 2.0)	0.695	0.217	linear
***L*. *vaginalis***	2.2 (1.3, 3.8)	0.003	2.4 (1.4, 4.3)	0.003	0.742	linear
***G*. *vaginalis***	0.3 (0.1, 0.7)	0.012	0.2 (0.1, 0.6)	0.004	0.769	binary
***A*. *vaginae***	0.2 (0.1, 0.5)	0.001	0.2 (0.1, 0.6)	0.001	0.745	binary
***G*. *vaginalis* cell-attached**	0.6 (0.4, 0.9)	0.015	0.5 (0.3, 0.8)	0.001	0.454	linear
***G*. *vaginalis* planktonic/dispersed**	0.6 (0.5, 0.8)	0.001	0.6 (0.5, 0.8)	0.001	0.815	linear
***A*. *vaginae* cell-attached**^**1**^	1.3 (0.6, 2.8)	0.572	0.5 (0.2, 1.2)	0.126	0.069	binary
***A*. *vaginae* planktonic/dispersed**	0.6 (0.2, 1.2)	0.149	0.2 (0.1, 0.5)	<0.001	0.052	binary
**Contraceptive vaginal ring**						
***Lactobacillus* genus**	1.3 (0.7, 2.2)	0.408	1.3 (0.8, 2.3)	0.310	0.874	linear
***G*. *vaginalis***	0.6 (0.3, 1.3)	0.169	1.1 (0.6, 2.2)	0.776	0.235	linear
***A*. *vaginae***	0.8 (0.4, 1.3)	0.307	0.8 (0.5, 1.2)	0.266	0.996	linear

^1^All, except one, were accompanied by cell-attached *G*. *vaginalis*

Note.-The qPCR results were expressed categorically as present or absent. The presence of species was modelled using mixed-effects logistic regression models and results were expressed as odds ratio (OR) of presence with 95% confidence interval (CI). When the model includes a binary time effect, the odds ratio is the odds of presence post-baseline divided by the odds of presence at baseline. When the model includes a linear time effect, the odds ratio is the odds of presence at a certain visit divided by the odds of presence at the previous visit.

The use of time as a categorical variable to a linear time effect and a binary time effect were compared and the most appropriate one was selected.

*Lactobacillus* genus (96.7%), *L*. *iners* (75.0%) and *G*. *vaginalis* (70.0%) were persistently present in the vaginal microbiota of the majority of women. The mean log_10_ geq/ml vaginal swab eluate of *Lactobacillus* genus and *L*. *iners* increased significantly (*Lactobacillus* genus both groups: *P*<0.001; *L*. *iners* intermittent: *P* = 0.013, continuous: *P* = 0.003), whereas that of *G*. *vaginalis* decreased significantly (both groups: *P*<0.001) over time (Table 3, S2 Fig).

**Table 3:** Longitudinal analysis of qPCR concentrations (log_10_ (geq)/mL of vaginal swab eluate) and Nugent scores in the Vagina and of qPCR concentrations (log_10_ geq/mL of vaginal ring eluate) and density of contraceptive vaginal ring biomasses in both study groups.

Dependent variable	Time evolution for intermittent use group		Time evolution for continuous use group		Difference in time evolution between groups	
	Estimate (95% CI)	P-value	Estimate (95% CI)	P-value	P-value	Time effect
Vaginal specimen						
*Lactobacillus* genus	0.8 (0.4, 1.1)	<0.001	0.9 (0.5, 1.2)	<0.001	0.671	binary
*L*. *iners*	0.7 (0.1, 1.2)	0.013	0.8 (0.3, 1.3)	0.003	0.755	binary
*G*. *vaginalis*	-1.2 (-1.8, -0.6)	<0.001	-1.3 (-1.9, -0.7)	<0.001	0.786	binary
Composite qPCR vaginal health score	2.1 (1.3, 2.8)	<0.001	2.3 (1.6, 3.0)	<0.001	0.681	binary
Nugent score	-1.6 (-2.3, -0.8)	<0.001	-1.9 (-2.7, -1.2)	<0.001	0.462	binary
Contraceptive vaginal ring						
*Lactobacillus* genus	-0.0 (-0.2, 0.1)	0.826	-0.0 (-0.2, 0.1)	0.657	0.868	linear
*G*. *vaginalis*	-0.3 (-0.5, -0.0)	0.024	—0.1 (-0.3, 0.1)	0.433	0.263	linear
*A*. *vaginae*	-0.1 (-0.4,0.1)	0.274	-0.2 (-0.4,0.0)	0.127	0.768	linear
Composite qPCR vaginal health score	0.3 (0.0, 0.6)	0.050	0.1 (-0.2, 0.4)	0.610	0.281	linear
Density	0.0 (-0.0, 0.1)	0.294	0.0 (-0.1, 0.1)	0.923	0.410	linear

Note.-The log_10_ concentrations (expressed in geq/mL vaginal swab eluate) of species that were persistently present in the vaginal microbiota, Nugent scores, vaginal and ring biomass composite qPCR vaginal health scores, and ring biomass densities were modelled with mixed-effects linear regression models. For ring biomass data baseline measurements were not applicable. Results were expressed as estimates (E) of change in mean log_10_ geq/mL vaginal swab eluate, in mean composite qPCR vaginal health score, in mean Nugent score or in mean biomass density with 95% confidence intervals (CI). When the model includes a binary time effect, E is the mean difference between post-baseline and baseline. When the model includes a linear time effect, E is the mean difference between two consecutive visits.

Effects of intermittent versus continuous use were tested as differences in slope in the models at the 5% level (two-sided).

Composite qPCR vaginal health scores were calculated as log_10_ (*Lactobacillus* genus)-log_10_ (*G*. *vaginalis*+ *A*. *vaginae*).

The mean composite qPCR vaginal health scores were significantly higher at the end of the study compared to the baseline values in both groups (Table 3). This effect did not significantly differ between the two groups.

**Fig 2:**
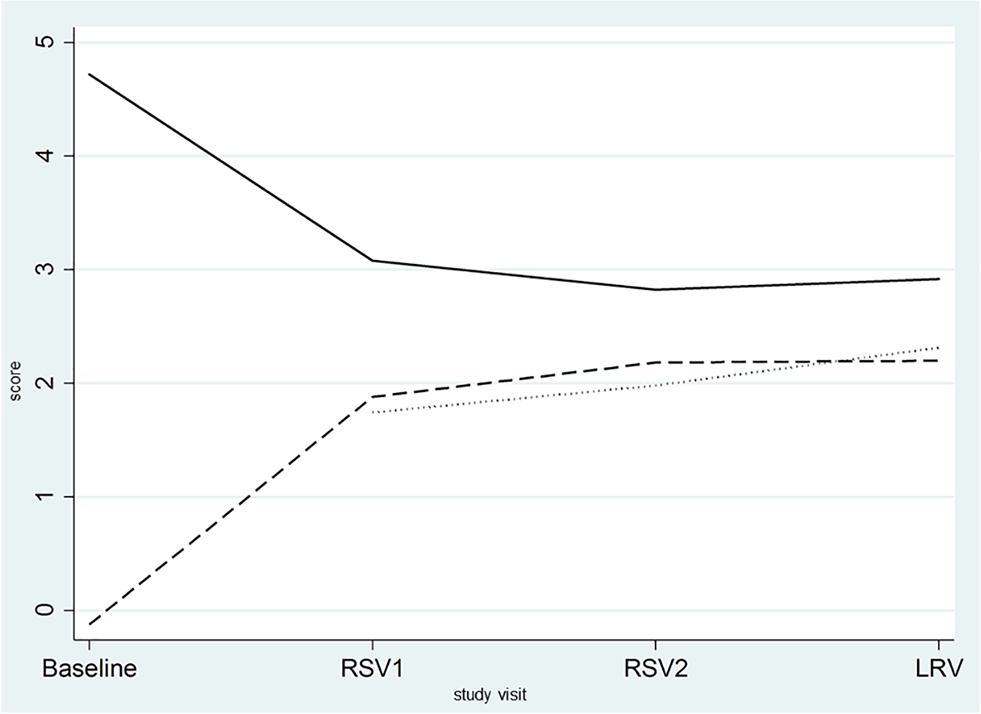
Evolution over time of the mean vaginal Nugent score, the mean composite qPCR vaginal health score of vaginal specimens, and the mean composite qPCR vaginal health score of contraceptive vaginal ring biomass. The Y axis is a score from 0 to 10 for both the Nugent and the composite qPCR vaginal health score. RSV1: first ring removal visit; RSV2: second ring removal visit; LRV: last ring removal visit ____: mean Nugent score _ _ _: mean composite qPCR vaginal health score of the vaginal specimens ……: mean composite qPCR vaginal health score of the contraceptive vaginal rings The composite qPCR vaginal health score is calculated as log_10_ (*Lactobacillus* spp.)-log_10_ (*G*. *vaginalis*+ *A*. *vaginae*).

The mean Nugent score decreased significantly over time (both groups: *P*<0.001) and did not differ significantly between intermittent and continuous users (Table 3 and Fig 2).

On average, the vaginal Nugent and composite qPCR vaginal health scores decreased and increased, respectively, after use of the first CVR and remained stable thereafter as shown in Fig 2.

The odds of vaginal biofilms comprising of cell-attached and dispersed *G*. *vaginalis* as identified using PNA-FISH decreased significantly over time in both study groups (cell-attached, intermittent: *P* = 0.015, cell-attached, continuous and planktonic/dispersed, both groups *P* = 0.001). The odds of presence of planktonic/dispersed *A*. *vaginae* significantly decreased in the continuous users only (*P*<0.001). The presence of biofilm containing *A*. *vaginae*, identified by PNA-FISH as attached to vaginal epithelial cells, did not significantly change over time (Table 2, S3 Fig). All vaginal biofilms containing *A*. *vaginae*, except one, contained *G*. *vaginalis* as well, but not vice versa.

### Biomass consisting of epithelial and bacterial cells accumulated on the CVR and its composition did not change over time

An adherent biomass was detected on all 415 collected CVRs with a mean optical density after CV staining of 3.35 (range 0.132–3.91). The mean biomass density did not evolve significantly over time in any one group neither did it significantly differ between the groups (Table 3).

Of the 415 collected CVRs, two CVR pieces for qPCR were missing. Bacteria were amplified from all 413 CVRs: 93% contained *Lactobacillus* genus, 57% *G*. *vaginalis* and 37% *A*. *vaginae*. Over the course of the study, the odds of presence of *Lactobacillus* genus, *G*. *vaginalis* and *A*. *vaginae* on the CVRs did not change significantly for either group (Table 2). However, the mean log_10_ geq/ml vaginal swab eluate of *G*. *vaginalis* decreased significantly in the intermittent (*P* = 0.024) and not in the continuous users (Table 3).

Applying the composite qPCR vaginal health score to the rings, a borderline significant increase of the score was observed for the CVRs used intermittently (*P* = 0.050) but not for those used continuously. However, the difference between the two groups was not significant (Table 3). The composite qPCR vaginal health scores of the CVRs and vaginal specimens were concordant and evolved similarly.

**Fig 3:**
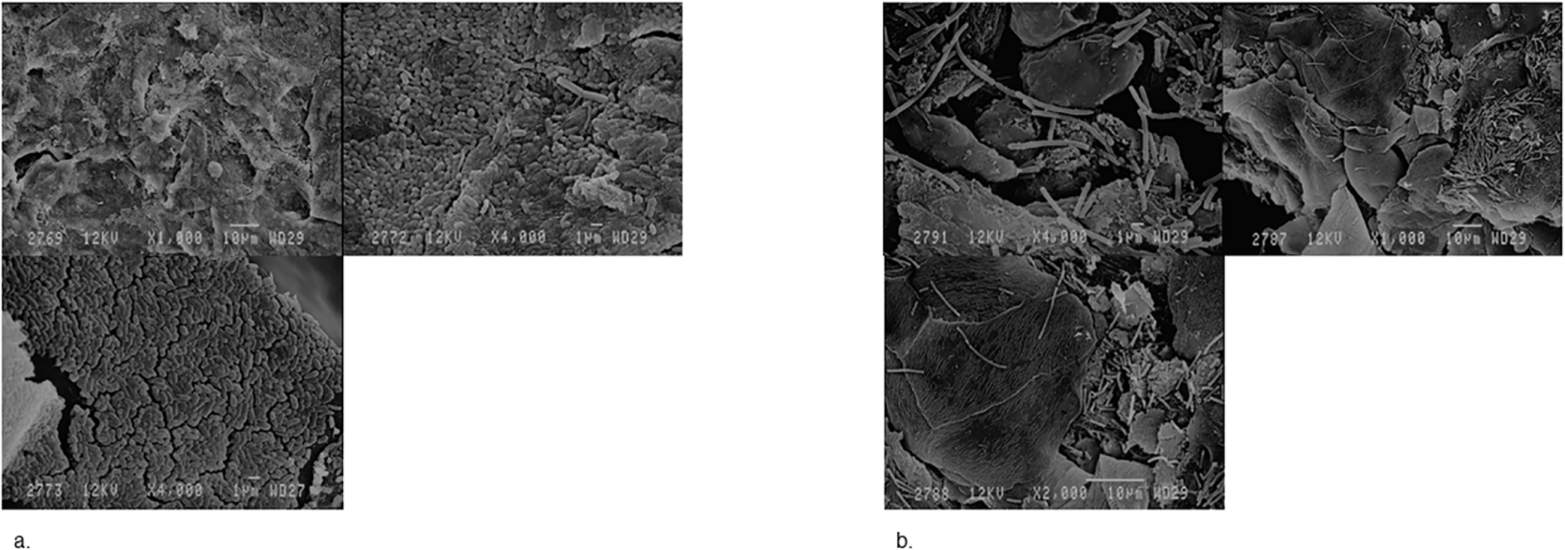
Scanning electron microscope observation of the biomass accumulated on contraceptive vaginal rings used for three weeks. Pictures 1a-3a: The contraceptive vaginal ring was used by a woman with a Nugent score of 10 and with presence of *Lactobacillus iners*, *Gardnerella vaginalis* and *Atopobium vaginae* as measured by qPCR. A vaginal biofilm consisting of *Gardnerella vaginalis* and *Atopobium vaginae* was identified using fluorescence in situ hybridization. On the ring *Lactobacillus species*, *Gardnerella vaginalis* and *Atopobium vaginae* were detected using qPCR, the crystal violet optical density was 3.7555 Pictures 1b-3b: Biomass on a contraceptive vaginal ring after use by a woman with a Nugent score of 0 and presence of *Lactobacillus iners* only. No biofilm was visualized using fluorescence in situ hybridization. On the ring *Lactobacillus species* was detected using qPCR, the crystal violet optical density was 3.7225.

SEM revealed that observed biomasses on the CVRs were composed of accumulated vaginal epithelial cells either covered by loose structures of scattered elongated bacteria with a morphology compatible with lactobacilli (Fig 3 pictures 1a-3a), or by dense structures compatible with a biofilm structure of coccobacilli type (Fig 3 pictures 1b-3b).

### The biomass on the CVR mirrored the vaginal microbiota

Four hundred seven CVR and vaginal specimen pairs were obtained, concordance as measured by qPCR (presence/absence) was observed in 94% of the pairs for *Lactobacillus* genus, 81% for *G*. *vaginalis*, and 88% for *A*. *vaginae*. Overall, the mean bacterial geq/mL vaginal ring eluate detected on the CVRs were 0.5 to 1.1 log_10_ lower compared to the mean geq/mL vaginal swab eluate in the vaginal secretions.

**Fig 4:**
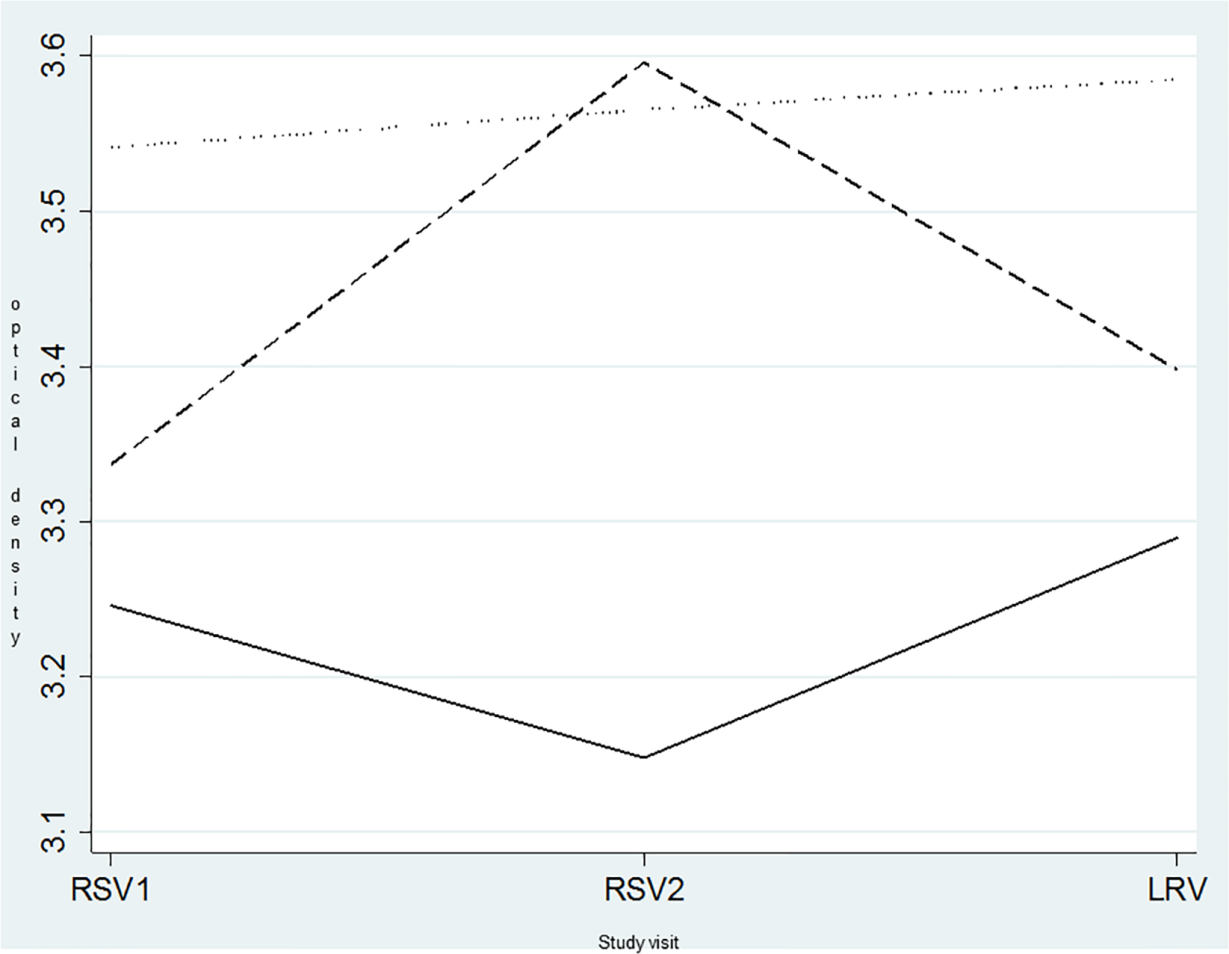
Evolution over time of the density of contraceptive vaginal ring biomass according to the normal, intermediate and BV vaginal microbiome as determined by Nugent scoring. The Y axis is the optical density of the crystal violet measurements of the contraceptive vaginal rings. RSV1: first ring removal visit; RSV2: second ring removal visit; LRV: last ring removal visit ____: mean optical densities of ring’s biomass collected from women with a normal vaginal microbiota according to the Nugent score (0–3) _ _ _: mean optical densities of ring’s biomass collected from women with an intermediate vaginal microbiota according to the Nugent score (4–6) ……: mean optical densities of ring’s biomass collected from women with bacterial vaginosis according to the Nugent score (7–10).

Fig 4 shows the evolution over time of the mean CVR biomass density using CV staining. Women with a BV Nugent score of 7–10 developed the densest CVR biomass with a mean optical density range of 3.5–3.6. Women with a normal Nugent score developed significantly less CVR biomass and the optical density fluctuated between 3.1 and 3.3. The largest fluctuation of the CVR biomasses optical density from 3.3 up to 3.6 was observed in women with an intermediate Nugent score of 4–6 (Fig 4).

## Discussion

We demonstrated that NuvaRing® use in a population with a high BV prevalence improves the vaginal microbiota over time. This observation was made independently of intermittent or continuous ring use regimen, and was defined by a decrease in mean Nugent scores and an increase in mean composite qPCR vaginal health scores (confirming the concurrent increase of lactobacilli and decrease of *G*. *vaginalis*) in both study groups. The composite qPCR vaginal health score evolution over time predicts whether the vaginal microbiota evolves towards a lactobacilli-dominated vaginal microbiota (increasing scores) or towards a dysbiosis (decreasing scores).

Furthermore, after the use of three CVRs, fewer women had a BV-associated vaginal biofilm containing *G*. *vaginalis*. However, we did not observe such a reduction in women who had a biofilm that also contained *A*. *vaginae*. Our findings agree with previous studies showing a favourable effect of CVRs on the vaginal microbiota [44–46], and are in line with earlier observations that the use of hormonal contraceptives, whatever their mode of administration, reduces the occurrence of BV [47,48]. These promising observations should be used to encourage the development of multipurpose interventions for the prevention of STIs and HIV in combination with pregnancy prevention.

Biomass was detected on all CVRs after having been inserted for three weeks, but biomass densities remained stable over time, independent of the CVR regimen used. The mean log_10_ concentrations (expressed in geq/mL vaginal ring eluate) of individual bacteria present in the ring biomasses did not significantly change over the course of the study either. A borderline increase of the mean composite qPCR vaginal health score, explained by the reduction over time of the mean *G*. *vaginalis* concentration, was observed in the intermittent users. However, the increase was minor and may be of no importance. The significant decrease of the mean vaginal Nugent score was achieved after the first CVR use where after the Nugent score stabilized. We also observed that the biomass on the CVR was associated with the vaginal Nugent score. Both observations explain the limited variation in biomass density over time. Details on the association of CVR biomass with vaginal Nugent score, dysbiosis and biofilm are presented elsewhere [38].

All cases, except for one, of vaginal biofilms including *A*. *vaginae* also included *G*. *vaginalis*, as observed by FISH analysis. The proportion of women with vaginal biofilms containing *G*. *vaginalis* decreased over time, but we could not show this for vaginal biofilms containing both *G*. *vaginalis* and *A*. *vaginae*. The presence of multiple bacterial species may strengthen the biofilm by specific multispecies interactions and gene regulations [49]. We postulate that, even though the exogenous oestrogen delivered by the CVR directly into the vagina increases the concentration of lactobacilli, the metabolites and bacteriocins produced by these lactobacilli are ineffective against the presence of a polymicrobial biofilm. Studies have indeed demonstrated that the robustness of biofilms increases with the diversity of its constituents [50]. However, we used FISH probes for *G*. *vaginalis*, *A*. *vaginae*, and total bacteria only and it is therefore possible that the biofilms containing *G*. *vaginalis* and not *A*. *vaginae* were polymicrobial nonetheless.

The quantity of biomass on the CVR was estimated using optical density measurement after CV staining. Unfortunately, CV also stains vaginal epithelial cells. We are therefore cautious in describing all biomass on the rings as biofilm. We hypothesize that vaginal epithelial cells play a major role in the attachment of the biomass on the vaginal ring and form a substrate layer for the vaginal microorganisms. Macromolecules, bacteria and tissue cells compete for space on the vaginal ring surface. It might be that epithelial cells adhere more rapidly to the ring surface than bacteria [51]. qPCR performed on the biomass revealed that for the majority of the participants the bacteria present on the rings were also present in their vaginal specimens. We did not determine whether the bacteria embedded in the biomass were alive, but in a study with Nesterone^®^/ ethinyl-estradiol CVRs used for up to one year, bacteria from the rings were grown, confirming their viability. The authors also reported a high level of vaginal-CVR agreement for *Lactobacillus* spp. and *G*. *vaginalis* [45].

Our continuous use regimen will most likely not reflect the way vaginal microbicides or multipurpose rings will be used in the future. The active product loadings in the NuvaRing^®^ is licensed for three weeks of use followed by a ring-free week, and off-label extended use may have increased the risk of unintended pregnancies. Our design of continuous use mimicked uninterrupted ring presence in the vagina for 12 weeks. However, individual CVRs were used for a maximum of three weeks in both regimens, which limited our ability to study biomass accumulation over extended periods of time.

We limited our study to the investigation of bacteria associated with vaginal health (several *Lactobacillus* spp) and bacterial vaginosis (*G*. *vaginalis* and *A*.*vaginae*). We did not include bacteria such as staphylococci, streptococci and bacteria belonging to the Enterobacteriaceae family that are associated with other types of VMC dysbiosis [52,53]. For now, we do not know whether contraceptive vaginal rings modify other dysbiotic VMC as well and whether biomass deposits on the CVRs also occur in the presence of other types of dysbiosis. We therefore recommend that future research in the context of vaginal health and vaginal ring use includes other types of dysbiosis as well.

In conclusion, NuvaRing^®^ promoted lactobacilli-dominated VMC in a population with high baseline BV prevalence despite the fact that biomass accumulated on the rings.

## Supporting information

S1 Checklist CONSORT checklist. (PDF)

S1 Protocol Final approved protocol for “The Ring Plus project: Safety and acceptability of vaginal rings that protect women from unintended pregnancy” version 2.0, 16 April 2013. (PDF)

S1 Fig Presence of *Lactobacillus* genus, *G*. *vaginalis* and *A*. *vaginae* in the vagina of the participants over the course of the study. The bar graphs present proportions of women with *Lactobacillus* genus, *G*. *vaginalis*, *A*. *vaginae* absent and present. The abbreviations RSV1, RSV2, RSV3 and LRV refer to the first ring removal visit, the second ring removal visit, the third ring removal visit, and the last ring removal visit, respectively.
(TIF)

S2 Fig Concentration of *Lactobacillus* genus, *G*. *vaginalis* and *A*. *vaginae* in the vagina of the participants over the course of the study. The boxplots depict the median (white line), 25th and 75th percentiles (box) of the mean log10 concentrations (expressed in genome equivalents/mL of vaginal swab eluate) of *Lactobacillus* genus, *G*. *vaginalis*, *A*. *vaginae* in vaginal swabs collected at baseline and the ring removal visits. The whiskers show the expected spread of the data, based on the median and interquartile range. Points outside of this range are individually indicated (possible outliers).
The abbreviations RSV1, RSV2, RSV3 and LRV refer to the first ring removal visit, the second ring removal visit, the third ring removal visit, and the last ring removal visit, respectively.
(TIF)

S3 Fig Presence of cell-adherent and dispersed/planktonic *G*. *vaginalis* and *A*. *vaginae* in the vagina of the participants over the course of the study. The bar graphs present proportions of women with cell-adherent *G*. *vaginalis*, dispersed/planktonic *G*. *vaginalis*, cell-adherent *A*. *vaginae*, dispersed/planktonic *A*. *vaginae* absent and present. The abbreviations RSV1, RSV2, RSV3 and LRV refer to the first ring removal visit, the second ring removal visit, the third ring removal visit, and the last ring removal visit, respectively.
(TIF)
