# Supplementary figures and images for: Contraceptive rings promote vaginal lactobacilli in a high bacterial vaginosis prevalence population: A randomised, open-label longitudinal study in Rwandan women
Source: PLoS One. 2018 Jul 23;13(7):e0201003. doi: 10.1371/journal.pone.0201003 (PMC6056036; doi:10.1371/journal.pone.0201003)

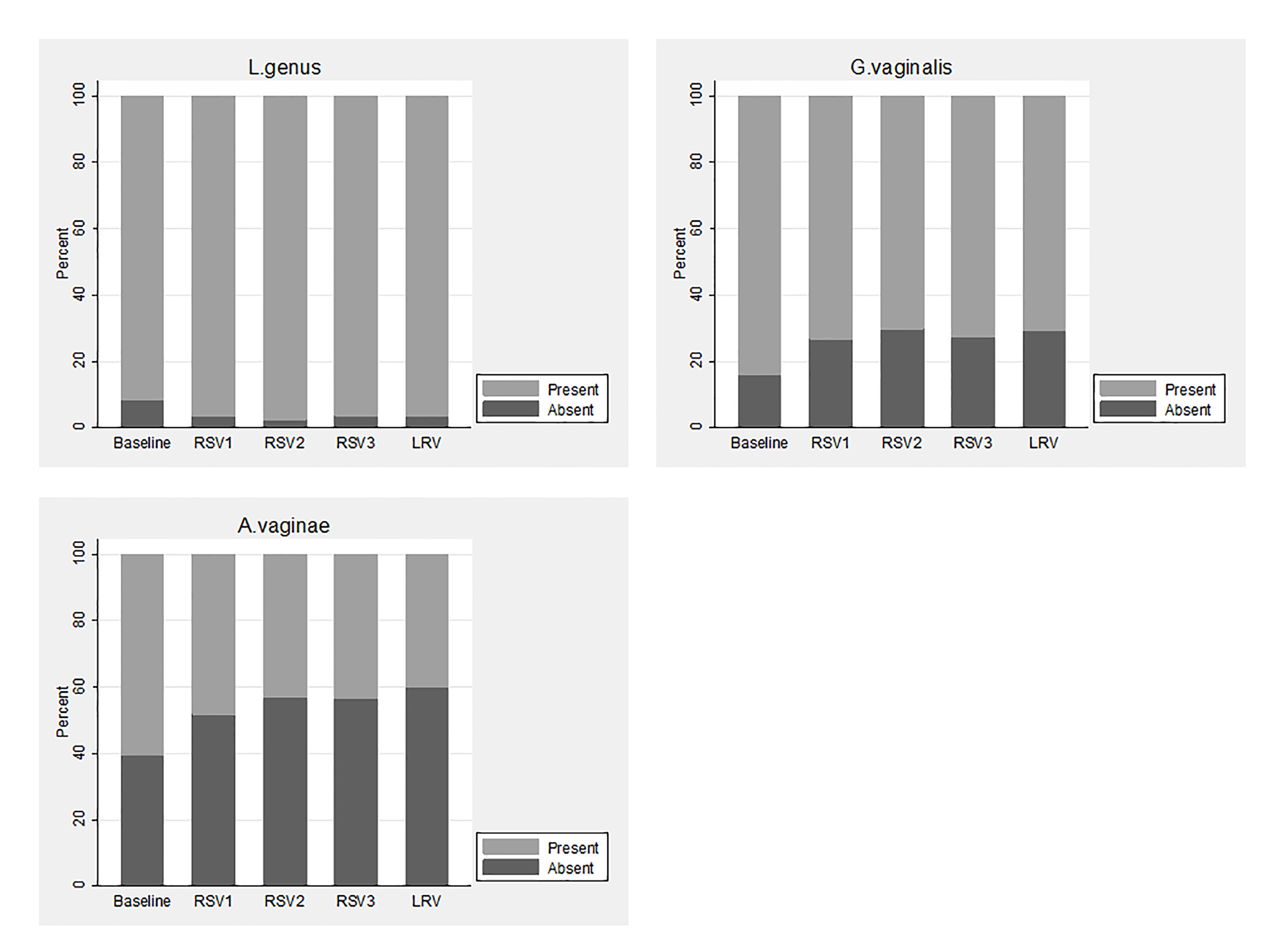

Supplement: S1 Fig — The bar graphs present proportions of women with Lactobacillus genus, G. vaginalis, A. vaginae absent and present. The abbreviations RSV1, RSV2, RSV3 and LRV refer to the first ring removal visit, the second ring removal visit, the third ring removal visit, and the last ring removal visit, respectively. (TIF) [file pone.0201003.s003.tif]

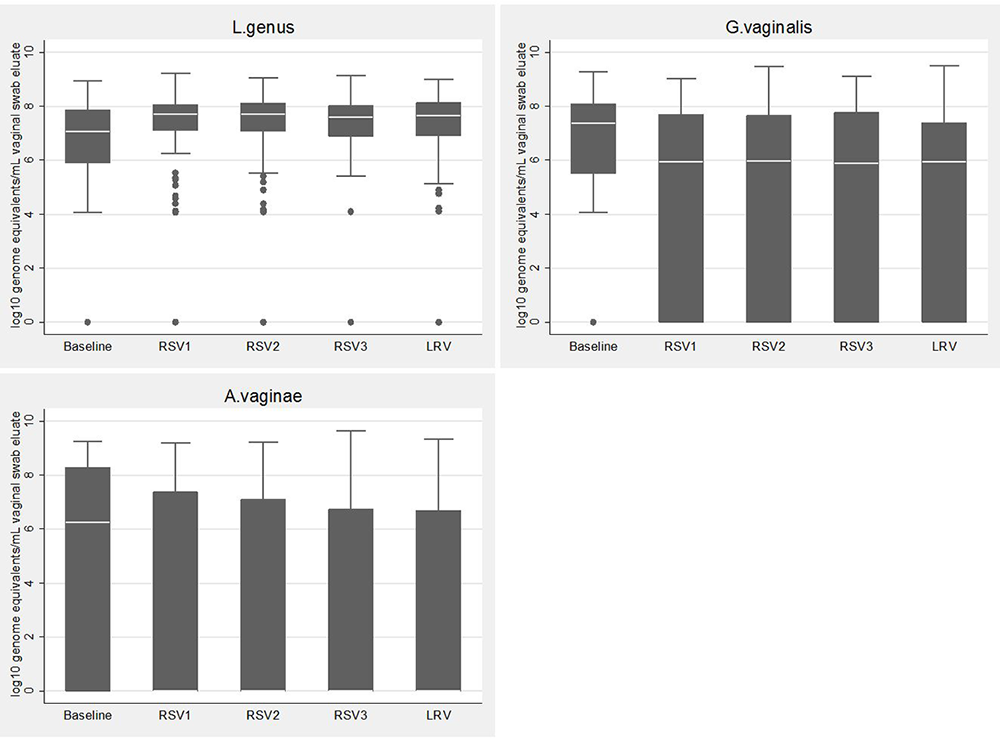

Supplement: S2 Fig — The boxplots depict the median (white line), 25th and 75th percentiles (box) of the mean log10 concentrations (expressed in genome equivalents/mL of vaginal swab eluate) of Lactobacillus genus, G. vaginalis, A. vaginae in vaginal swabs collected at baseline and the ring removal visits. The whiskers show the expected spread of the data, based on the median and interquartile range. Points outside of this range are individually indicated (possible outliers). The abbreviations RSV1, RSV2, RSV3 and LRV refer to the first ring removal visit, the second ring removal visit, the third ring removal visit, and the last ring removal visit, respectively. (TIF) [file pone.0201003.s004.tif]

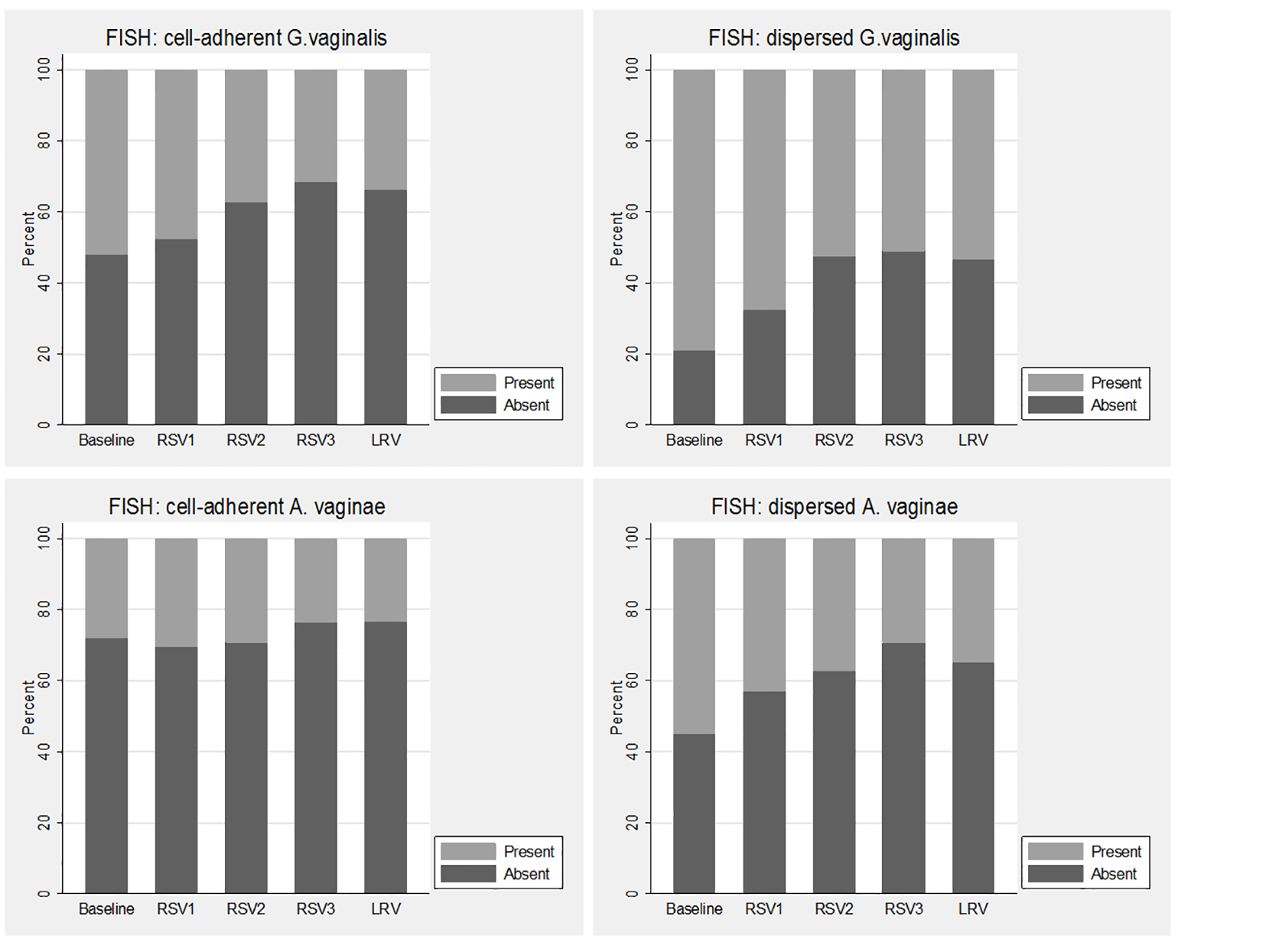

Supplement: S3 Fig — The bar graphs present proportions of women with cell-adherent G. vaginalis, dispersed/planktonic G. vaginalis, cell-adherent A. vaginae, dispersed/planktonic A. vaginae absent and present. The abbreviations RSV1, RSV2, RSV3 and LRV refer to the first ring removal visit, the second ring removal visit, the third ring removal visit, and the last ring removal visit, respectively. (TIF) [file pone.0201003.s005.tif]
